# A novel bi-planar calibration method for digital templating in total hip arthroplasty

**DOI:** 10.1038/s41598-023-28048-7

**Published:** 2023-01-25

**Authors:** Christian Ries, Tim Rolvien, Frank Timo Beil, Henriette S. Boese, Christoph Kolja Boese

**Affiliations:** 1 Division of Orthopaedics, Department of Trauma and Orthopaedic Surgery, University Medical Center Hamburg-Eppendorf, Hamburg, Germany; 2 Clinic for Anesthesiology and Operative Intensive Care, Albertinen Hospital, Martinistr. 52, 20246 Hamburg, Germany

**Keywords:** Skeleton, Computational science, Medical research

## Abstract

In total hip arthroplasty and reconstructive orthopedic surgery, pre-operative digital templating is essential for surgical treatment optimization, risk management, and quality control. Calibration is performed before templating to address magnification effects. Conventional methods including fixed calibration factors, individual marker-based calibration and dual-scale marker methods are not reliable. A novel bi-planar calibration method is described aiming to reduce the error below clinical significance. The bi-planar calibration method requires two conventional orthogonal radiographs and a standard radiopaque marker ball. An algorithm computes the hip plane height parallel to the detector in the antero-posterior radiograph. Foreseeable errors (i.e., patient rotation and misplaced markers or lateral offset) are considered in a correction algorithm. Potential effects of errors are quantified in a standard model. Influence of rotation in lateral radiographs and lateral offset of marker on the calibration factor are quantified. Without correction, patient rotation in the lateral radiograph of 30° results in absolute calibration error of 2.2% with 0 mm offset and 6.5% with 60 mm lateral offset. The error is below the threshold of 1.5% for rotation less than 26° with 0 mm offset and 10° with 60 mm offset. The method is supposed to be reliable in precisely predicting the hip plane and thereby the calibration factor. It may be superior to other methods available. In theory, the method allows correction of clinically relevant rotation of at least 30° and marker displacement without impacting the computed calibration factor.

## Introduction

**Figure 1:**
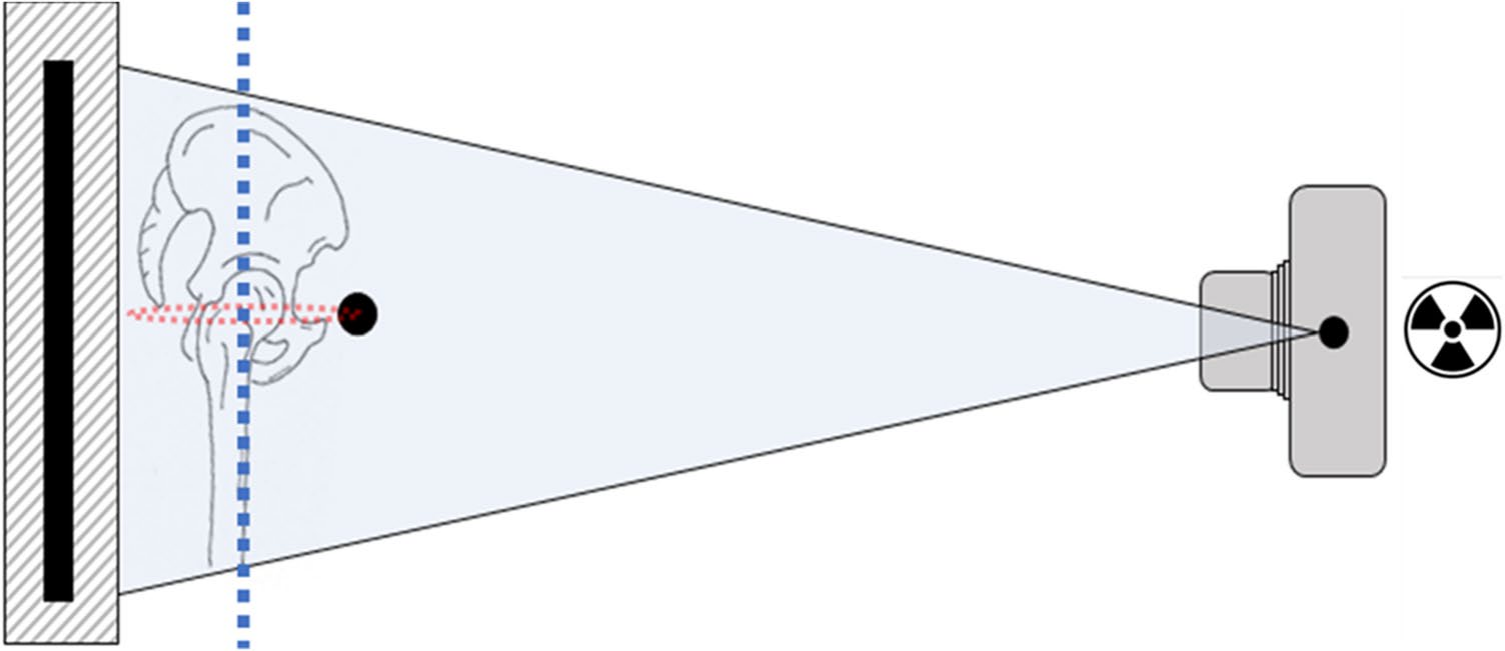
Cross-sectional view on setup of a.p. radiograph. Left area represents standing wall buckey and black line is the detector plane. Pelvis and femur of patient facing x-ray source (on the right). x-Ray beam shown. Blue dotted line represents hip plane as parallel plane to the detector plane through the hip centers.

In the United States, over 450,000 total hip replacements (THR) are performed annually, and the numbers are predicted to increase significantly in the future^1^. Pre-operative digital templating is considered the standard for THR and reconstructive surgery^2,3^. It is part of established risk management and quality control measures in arthroplasty. Templating of hip surgery is performed in digital antero-posterior radiographs of the pelvis. Here, the magnification of the radiograph needs to be taken into account and radiographs are calibrated^2,4–8^. As objects are magnified anti-proportional with the distance from the x-ray source, calibration aims to calibrate a specific distance from the x-ray source. This distance is represented as a plane parallel to the detector surface. In hip surgery, the plane of interest is the hip plane. This plane is the plane cutting through the center of both hip joints in antero-posterior radiographs of the pelvis (Fig. 1). Currently, there are three ways to achieve calibration: (1) application of a fixed calibration factor^4,7^, (2) individual marker-based calibration^4–6,8,9^ and (3) dual-scale marker methods^4,10,11^.

Fixed calibration factor methods are considered outdated and not reliable^9^. They do not take the individual hip plane position into account and thereby have an inherent error. The mean absolute error of the method has been shown to be 1.8% (1.6% and 2.0%) with a maximum error of 12.4%^4,10^.

Marker-based calibration requires automated, semi-automated or manual identification of the marker and its dimensions in the projected radiograph. Subsequently, the calibration factor is computed by the appropriate templating software.

The marker-ball method is the current clinical standard. By placing a spherical radiopaque marker of known size at the supposed height of the hip plane, the magnification of the radiograph can be calibrated^6,7,12^. However, this method is prone to misplacement of the marker and various studies found the error of the method to be unacceptable with mean errors of 8.4% (5.2–12.5) and maximum errors of up to 26.6%^4–6,8,9,13^.

Dual-scale calibration is a less common method^4,10,11^. Here, the sagittal diameter of the patient is measured in radiographs and the hip plane position is calculated based on empirical regression models. This method has been shown to have a mean absolute error of 1.1% (maximum 5.6%) and 2.2% (maximum 9.5%) in supine and standing radiographs, respectively^4,14^.

As the conventionally used intercept theorem is not appropriate for calculating calibration factors of spherical markers^6,12^, more complex formulae (i.e. Formula 1 and 2) are used that require computing by software.

Together, it can be concluded that none of the current methods is sufficiently reliable in calibrating radiographs for templating and planning and alternatives are sought^15^. Notably, per calibration error of about 3% (± 1.5%) a deviation of one component size from the optimal size may result in THR^16^. Optimal calibration should achieve reliable calibration errors of equal to or less than 1.5%^16^.

In this technical note, a novel, bi-planar calibration method is described with mathematical formulas, algorithms, and images in the methods section. Potential limitations are identified and analyzed. The main goal of the note is the description and application of the method, represented in the methods section. A simulation model highlights key limitations and quantifies possible effects addressed by the correction algorithm.

This method may be of particular interest for future integration of digital templating being directly translated into navigated and robotic-assisted surgery.

## Methods

No human subjects were involved in the present study.

### Clinical application, marker placement and radiographs

In a clinical setting, the bi-planar calibration method requires two (approximately) orthogonal radiographs of the pelvis with a single external calibration marker (ECM) in place: (1) a standard standing antero-posterior (a.p.) (low) radiograph of the pelvis. The beam is centered to the pubic symphysis and (2) a lateral standing radiograph of the pelvis.

The marker ball is fixed to the patient in the midline at the height of the pubic symphysis in a position approximately at the pubic symphysis (“belt-buckle position”). Fixation may be achieved with belts or other fixation devices. The belt-buckle position has been shown to be easy to identify and more comfortable for patients during marker placement compared to conventional positions between the legs^4,14^.

**Figure 2:**
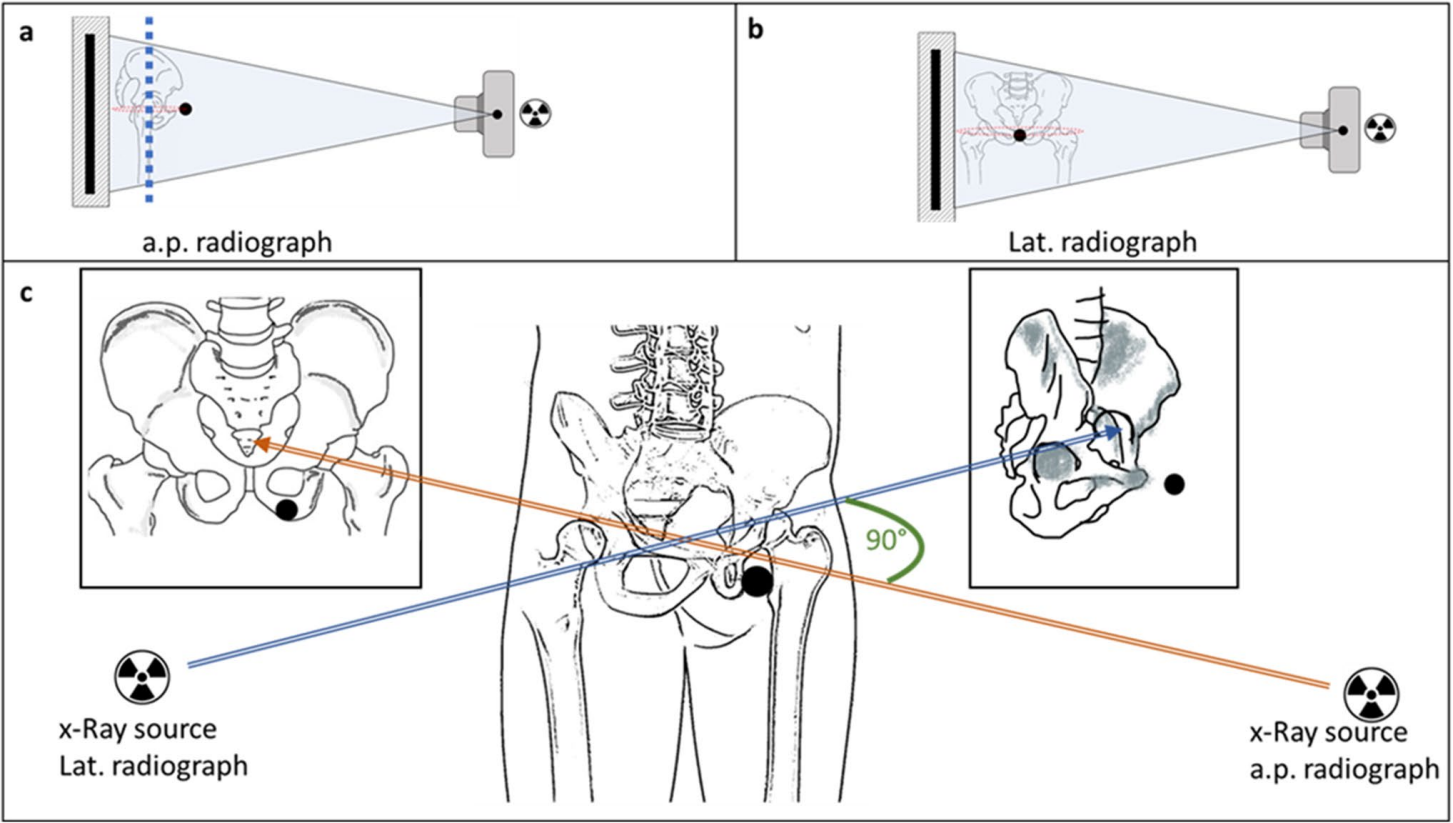
X-ray source, beam and detector in wall bucky are depicted in Fig. 2a,b. Dotted circle represents belt with marker (filled black circle). (**a**) Patient position for a.p. radiograph. Dotted line represents hip plane parallel to the detector plane. (**b**) Patient position for lateral radiograph. (**c**) Spatial representation of setup with resulting radiographs. Radiographs orthogonal to one another. *Note:* the resulting lateral radiographs is slightly rotated from strict lateral position to demonstrate use error in the following sections. Additionally, the marker position is placed with offset to visualize the effects and measurements in c, while being in the optimal position in a and b.

The marker stays in the same place relative to the patient during both radiographs (Fig. 2a–c). The order of radiographs taken is not relevant. While a true lateral view centered to the hip centers may not be achieved, the method is supposed to be robust and correct for potential inexact imaging. No special x-ray machine is required.

Finally, measurements and calculations of the proposed method could be integrated and automatized within existing templating software. Alternatively, a dedicated software application could be developed to identify the calibration factor which would be entered into conventional templating software either manually or automatically.

Therefore, the person performing templating would neither need to change the templating process nor be aware of the improved calibration method at all.

Symbols and terms used in the figures and the text are defined in Table 1.

**Table 1:** Definition of symbols and terms.

Automated or semi-automated measurements	
Landmarks lateral radiograph	Landmarks antero-posterior radiograph
* A* = central beam/center of image	* O* = central beam/center of image
* B* = Center of ECM	* P* = Center of ECM
* C* = Center of right hip	* Q* = Center of right hip
* D* = Center of left hip	* R* = Center of left hip
* E* = cutting point of *s* and *t*	* V* = mid between T and R
* G* = mid between *E* and *D*	* T* = cutting point of* o* and *n*
* H* = cutting point of *r* and *u*	* U* = cutting point of* w* and *p*
Lines lateral radiograph	Lines antero-posterior radiograph
* q* = line through* A* and *B*	* v* = line through* O* and *P*
* r* = horizontal line through *B*	* o* = horizontal line through *R*
* s* = vertical line through *C*	* n* = vertical line through *Q*
* t* = horizontal line through *D*	* w* = horizontal line through *P*
* u* = vertical line through *G*	* p* = *vertical line through* V
Variables lateral radiograph	Variables antero-posterior radiograph
* a* = direct distance Image-center to ECM center	* h* = direct distance Image-center to ECM center
* b* = long half-axis ECM diameter	* i* = horizontal distance Image-center to ECM center (i.e. lateral offset)
* c* = horizontal distance right hip center to left hip center	* k* = long half-axis ECM diameter
* d* = distance Horizontal hip center to ECM center	* m* = distance right hip center to left hip center
Calculated variables	Subscript indicates
* F*_*ap*_ = calibration factor in a.p. radiograph	Cal = calibrated variable
* F*_*lat*_ = calibration factor in lateral radiograph	Lat = lateral image derived
* F*_*Hip*_ = calibration factor of the hip plane in a.p. radiograph	Ap = antero-posterior image derived
* H*_*ECM*_ = height of ECM over detector	Correction = rotation corrected value
* H*_*Hip*_ = height of hip plane over detector	
n = number of interations (starts with zero)	
x = correction of *d* for *i* in lat. radiogprah	
y = calculated distance of ECM from inter-hip center	
α = rotation angle of patient in lateral radiograph	
Pre-defined variables	
* r* = radius of ECM (mm)	
* S* = source-detector-distance (mm)	

### Image analysis

**Figure 3:**
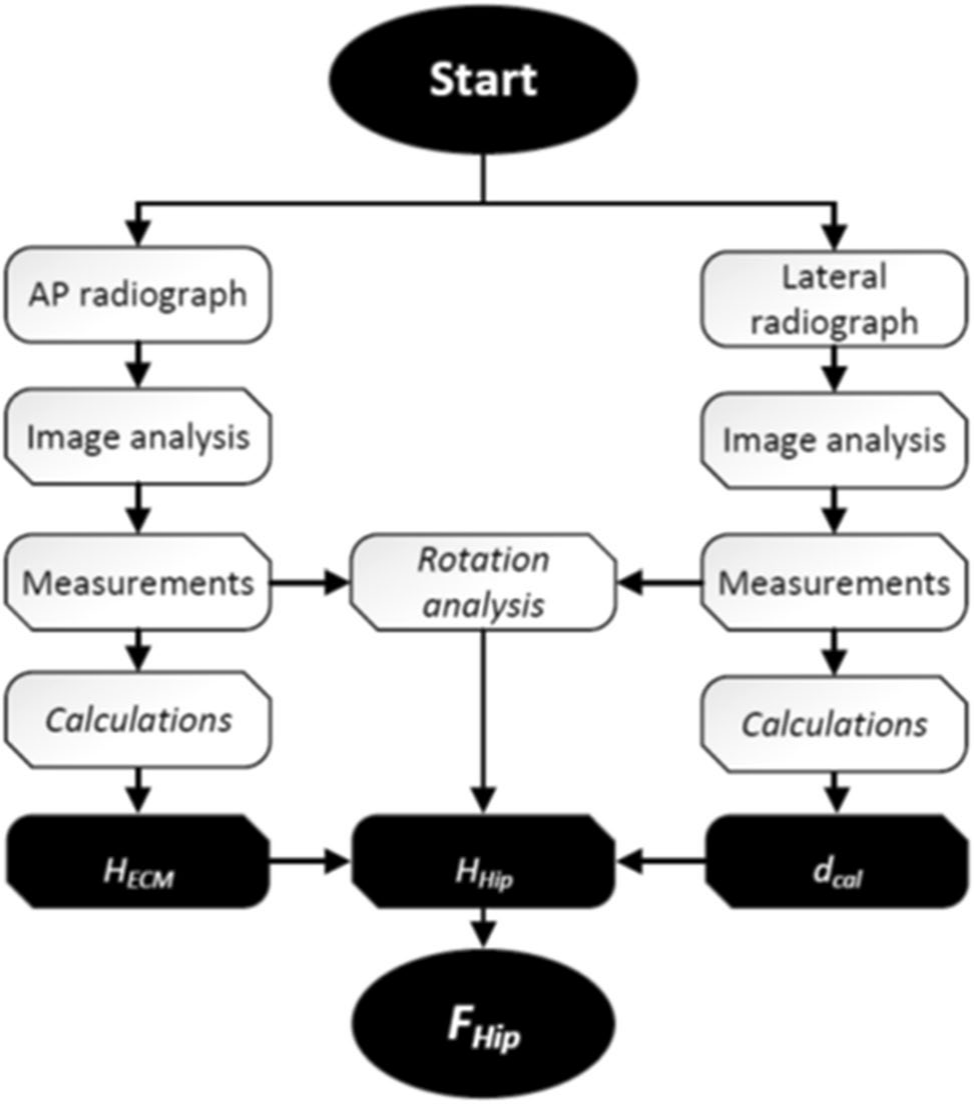
Algorithm overview of image analysis and calculation of the calibration factor of the hip plane (*F*_*Hip*_).

Measurements and calculations described hereby may be automated or semi-automated within autonomous software or integrated into templating software. Figure 3 presents the algorithm for computational calculation of the key variable: the calibration factor of the hip plane (*F*_*Hip*_).

As shown in Fig. 3, three major workflows may be distinguished. The calculation of the calibration factors (*F*) of the ECM in either image with the output of two variables for subsequent use (i.e. *H*_*ECM*_ and *d*_*cal*_). Additionally, the rotation of the patient and thereby the hip plane in the lateral radiograph is identified and quantified using data from both radiographs. This information is used to correct calculations from the a.p. radiograph and finally merge the information from both image analyses into the primary outcome parameter, the calibration factor of the hip plane (*F*_*Hip*_) as described in more detail in the following sections.

### Image analysis—identification of landmarks and objects, lines and distances

**Figure 4:**
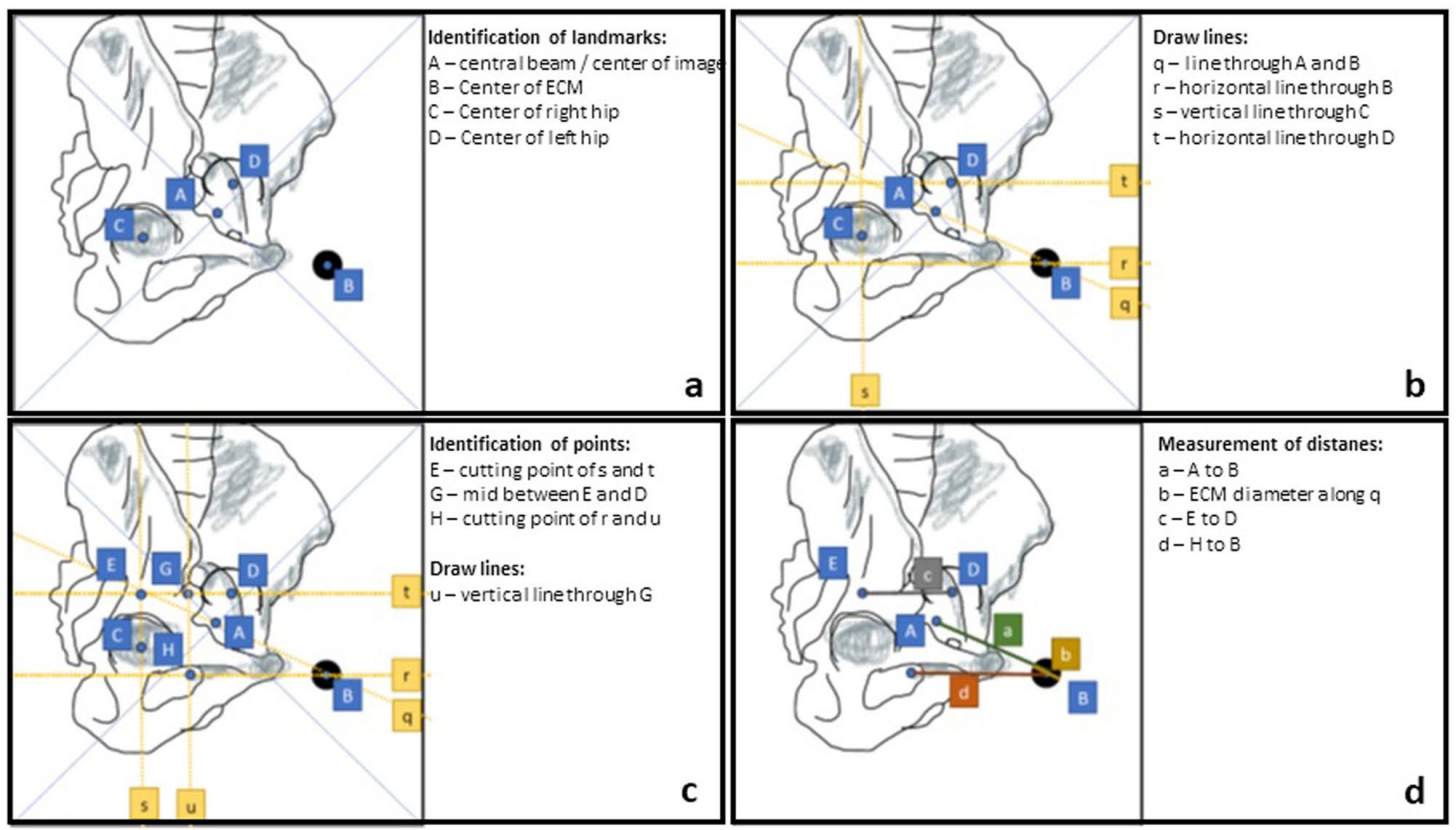
Image analysis in the lateral radiograph. Note: the pelvis is slightly rotated to ease identification of landmarks and measurements in a non-optimal lateral radiograph. (**a**) Identification of landmarks/points. (**b**) Drawing of lines defined by landmarks. (**c**) Identification of additional cutting points and drawing of additional lines. (**d**) measurement of key distances.

**Figure 5:**
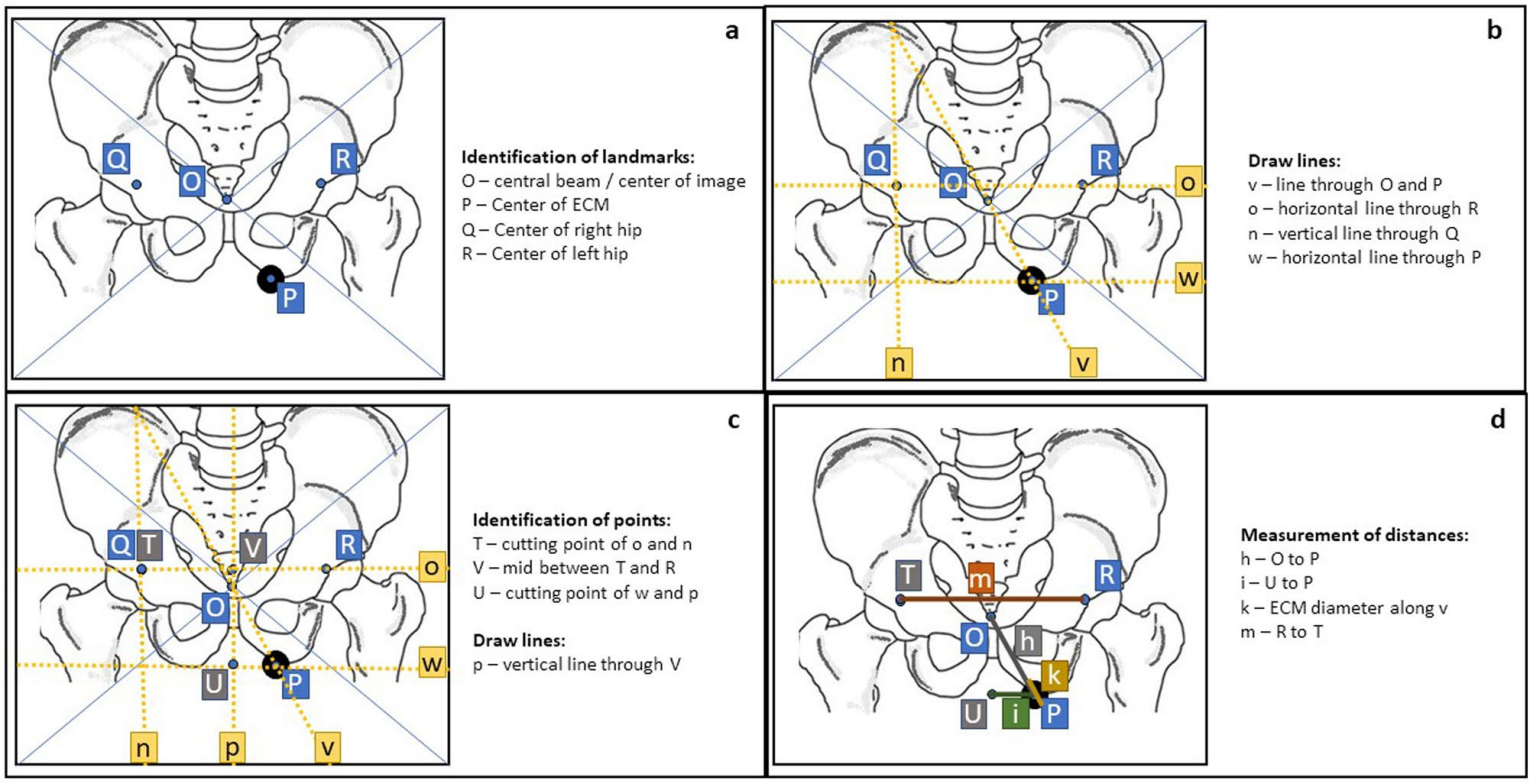
Image analysis in the a.p. radiograph. Note: the marker position is placed with offset to visualize the effects and measurements. (**a**) Identification of landmarks/points. (**b**) Drawing of lines defined by landmarks. (**c**) Identification of additional cutting points and drawing of additional lines. (**d**) measurement of key distances. Note: *Q* and *T* may be very close or in the same place depending on the patient position and anatomy.

Figures 4 and 5 show sketches of lateral and antero-posterior radiographs, respectively. Here, the relevant measurements are outlined, and symbols/variables defined in Table 1 are visualized.

**Figure 6:**
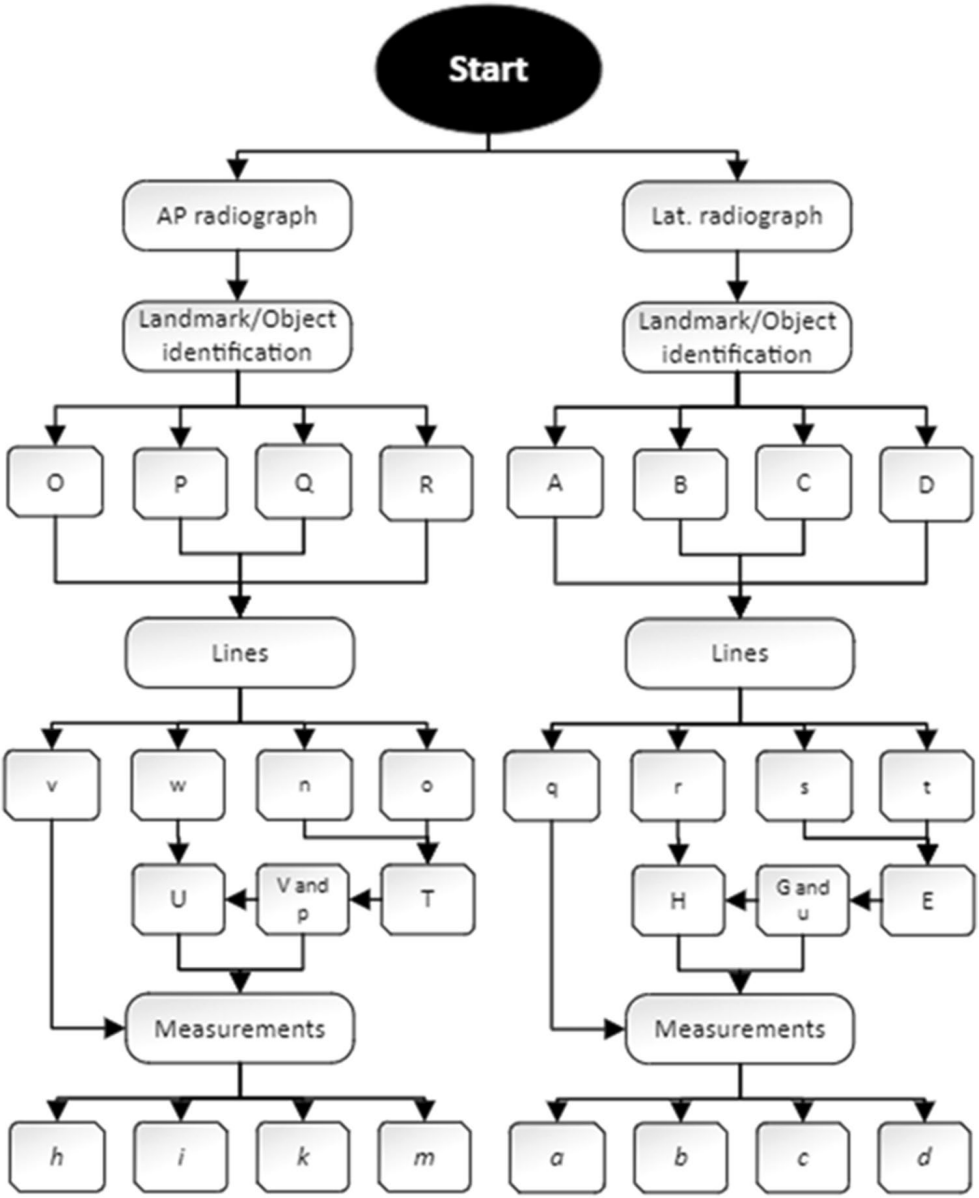
Algorithm of a.p. and lateral radiograph analysis.

The algorithm of the measurements is depicted in Fig. 6.

### Calculation of calibration factors, H_ECM_ and d_cal_

In both radiographs, antero-posterior and lateral, the vertical height of the ECM above the detector and thereby the calibration factors (*F*_*lat*_ and *F*_*ap*_) are calculated according to the method described by Boese et al. (Formula 1 and Formula 2 for *F*_*lat*_ and *F*_*ap*_, respectively)^6,12^. Here, *r* is the radius of the ECM in mm and *S* is the source-detector-distance of the x-ray device setup in mm.

1 $$F_{lat} = S - \sqrt {r^{2} \left( {1 + \frac{{2S^{2} }}{{b^{2} }}} \right) + \frac{2rS}{b}\sqrt {\frac{{r^{2} S^{2} }}{{b^{2} }} + a_{0}^{2} } } .$$

2 $$F_{ap} = S - \sqrt {r^{2} \left( {1 + \frac{{2S^{2} }}{{k^{2} }}} \right) + \frac{2rS}{k}\sqrt {\frac{{r^{2} S^{2} }}{{k^{2} }} + h_{0}^{2} } } .$$

The projected lateral displacement of the ECM (*a* and *h*) from the vertical hip center line (*s* and *u*) in any image is considered in the calculation of the lateral and antero-posterior calibration factors (*F*_*lat*_ and *F*_*ap*_). To take the calibration of the projected distances into account, the computational algorithm undergoes repeated iterations of the calculation of the calibration factors until the latest calibration factor differs less than 0.0001 from the previous calculation (e.g. *F*_*ap*_^*n−1*^ − *F*_*ap*_^*n*^ < 0.0001, for n being the number of iterations). There must be at least one iteration. The pre-defined difference may be changed from 0.0001 depending on required precision.

**Figure 7:**
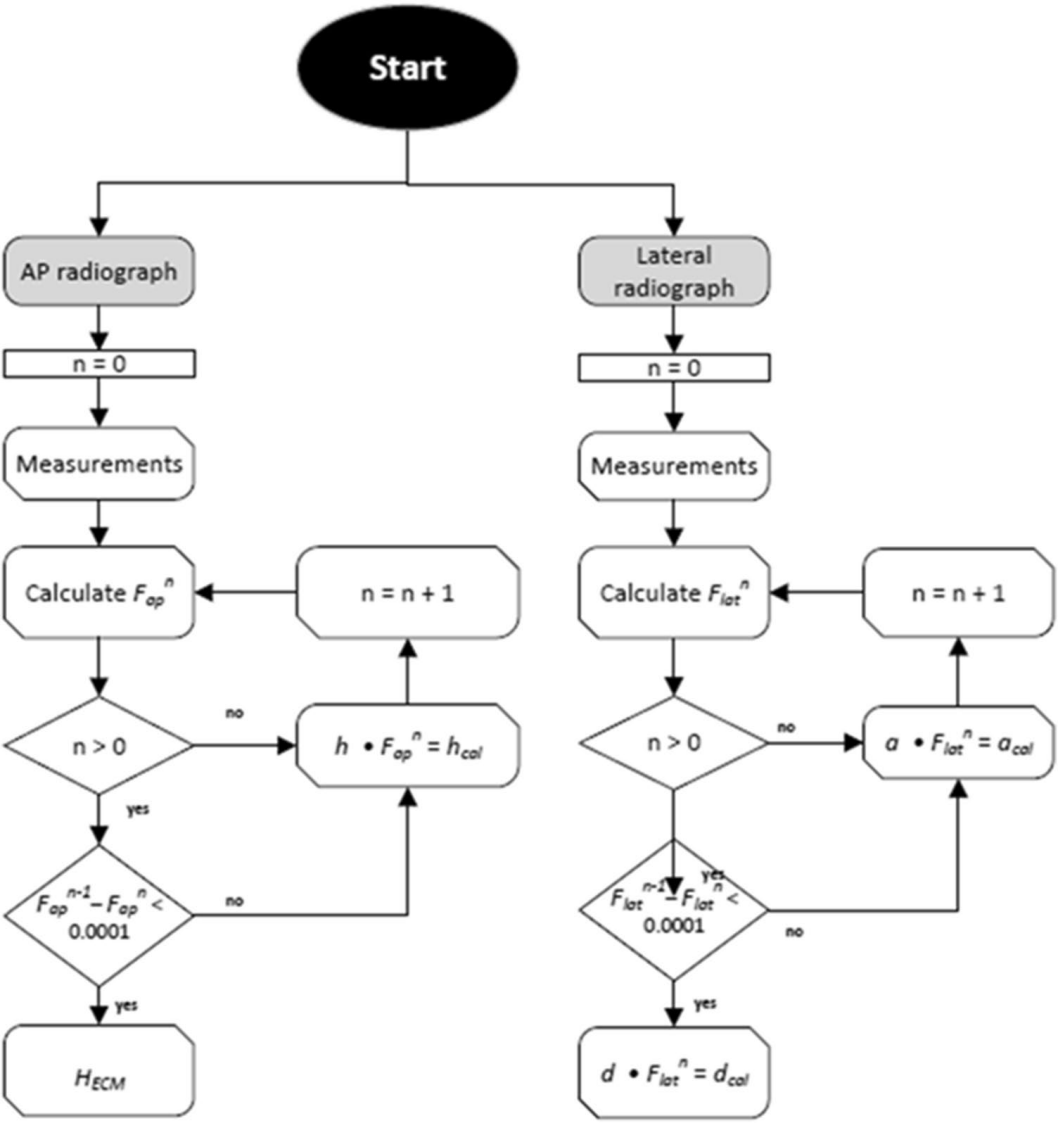
Algorithm for a.p. and lateral radiograph analysis with multiple iterations.

The algorithm is outlined in Fig. 7.

The output of the a.p. radiograph is the height of ECM over the detector (*H*_*ECM*_). The output of the lateral radiograph is the height of ECM over the hip plane (*d*_*cal*_).

### Use errors: patient rotation and marker misplacement

To calculate *H*_*ECM*_, two use errors may be addressed with computational calculations:

- Misplacement of the marker resulting in lateral deviation from the center of the hips. This effect is called lateral offset of the marker.
- Patient rotation from the strict lateral position during lateral radiographs resulting in oblique imaging.

**Figure 8:**
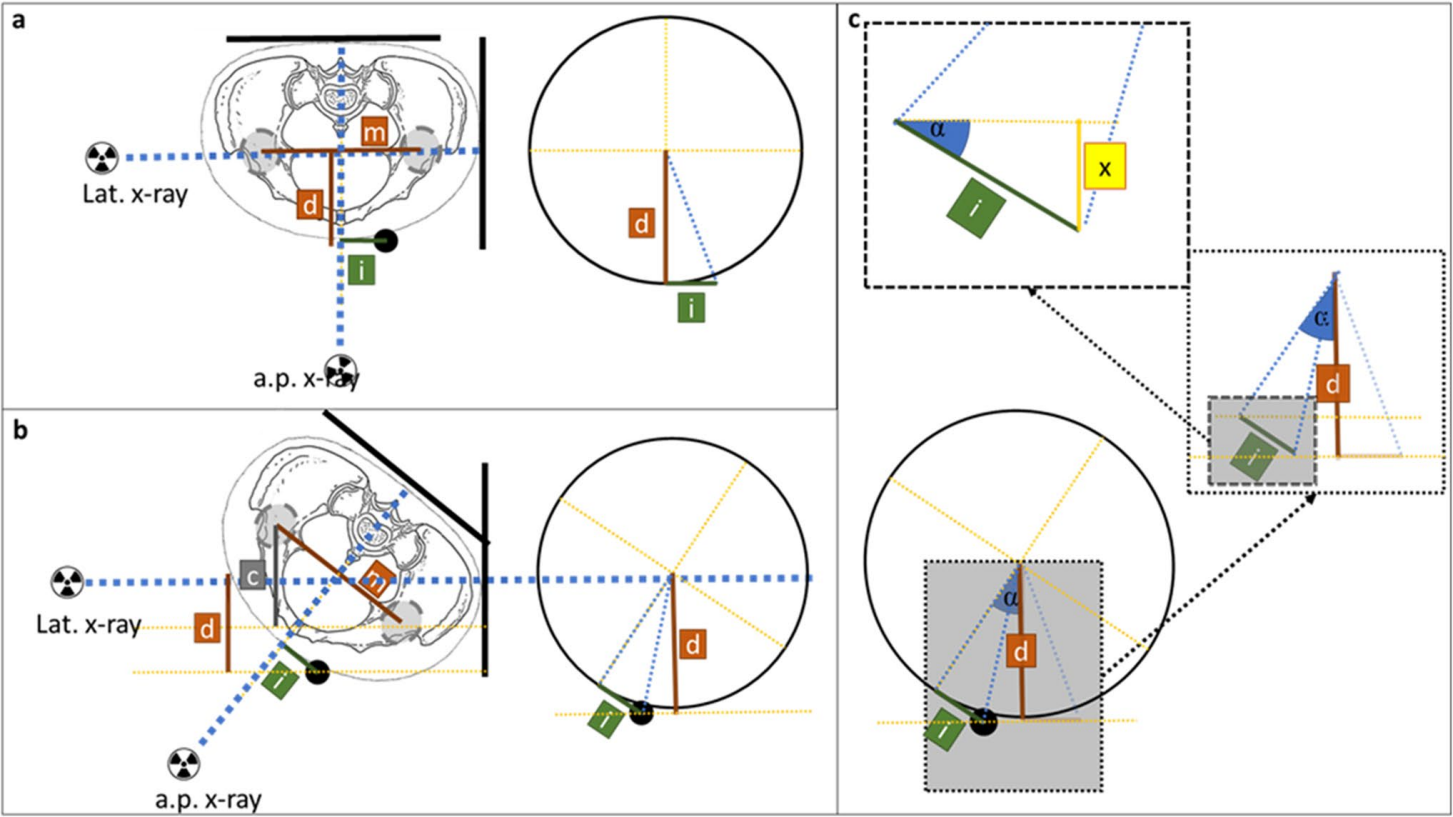
Effects of patient rotation and lateral offset. Top view on patient. Pelvis depicted. Bold black line presents detector plane. X-Ray sources for lateral and a.p. radiograph presented. Dotted blue line represents central beam. (**a**) Optimal orthogonal radiographs without rotation. (**b**) Rotated patient in lateral radiograph. Lateral offset *i* affects projection of *d*. (**c**) Rotation angle α affects *d* and *i*. Projection of *d* is shortened or lengthened by *x*. *x* can be calculated using α and *i*.

In Fig. 8 the combined effects of marker misplacement and patient rotation in the lateral radiographs are depicted.

### Identification of use errors in radiographs

**Figure 9:**
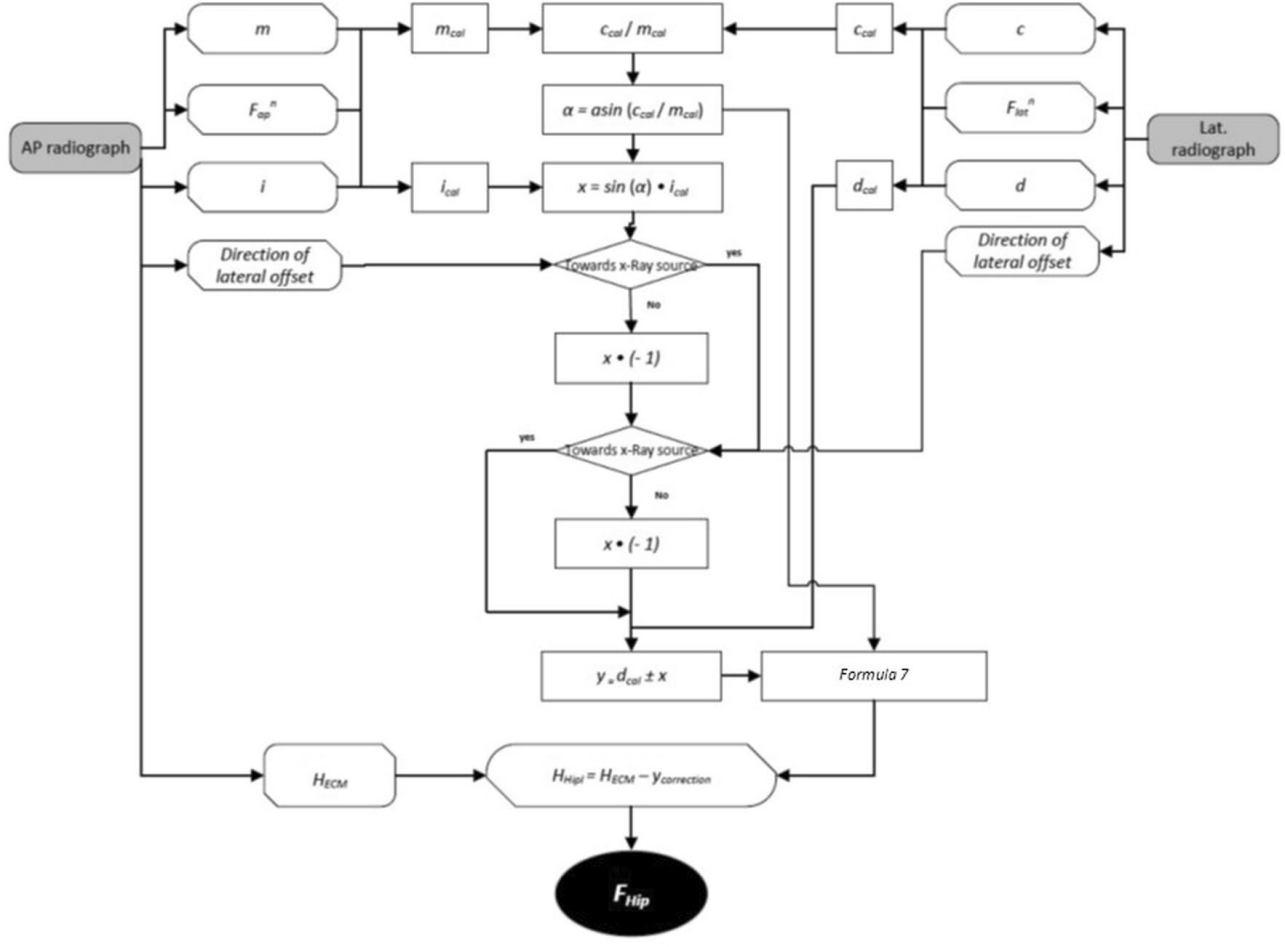
Algorithm for rotation correction.

Misplacement of the marker is identified in the a.p. radiograph as any horizontal deviation of the ECM center from the central beam (*i* > 0). It is of relevance in the algorithm for the correction of patient rotation in the lateral image (Fig. 9). Here, the direction of the lateral offset is of relevance. The direction of the lateral offset to the right or left side of the patient in the a.p. radiograph will define in whether the offset it towards the source or the detector plane in the lateral radiograph depending on the direction the patient faces towards in the lateral radiograph (looking left or right). The direction of the lateral offset per definition is defined in Table 2. For example, lateral offset of the ECM towards the right side of the patient will result in offset towards the source in case the patient faces to the left (contralateral) direction during the lateral radiograph.

**Table 2:** Direction of lateral offset in lat. radiograph.

Direction of lateral offset in a.p, radiograph	Direction patient faces in lateral radiograph (relative to x-ray source	
	Right	Left
Right	Away from source	Towards source
Left	Towards source	Away from source

Patient rotation during the lateral radiograph is identified by imperfect overlay of the two hip centers. Based on the calibrated distance of the hip centers from one another in a.p. radiograph (*m*_*cal*_) and the lateral radiograph (*c*_*cal*_), the rotation of the patient (α) can be assessed by Formula (3):

3 $$\alpha = {\text{asin}}\left( {\frac{{C_{cal} }}{{m_{cal} }}} \right).$$

### Correction for patient rotation and lateral offset

The projected length of *d* is influenced by the rotation α as well as the lateral offset *i*. Depending on the rotation of the patient (from or towards the source) and the direction of the lateral offset, the projection of *d* is shortened or lengthened by *x* in the lateral radiograph. Here, *x* is the opposite the hypotenuse *d* and the rotation angle α as shown in Fig. 8.

While it cannot be measured in the lateral radiograph, the length of *i*_cal_ is known from the a.p. radiograph. *x* can be calculated following Formula (4):

4 $$x = \sin \left( \alpha \right) \times i_{cal} .$$

Depending on aforementioned direction of rotation and lateral offset, the value of *x* may be positive or negative; *x* is multiplied by − 1 for offset away from the source and + 1 by offset to the source; *x* is multiplied by − 1 for rotation away from the source and + 1 by rotation to the source.

By addition of *d*_cal_ and *x*, the projection of *d*_cal_ without the lateral offset (*y*) is calculated.

5 $$y = d_{cal} \pm x.$$

The rotation corrected length of *y* (*y*_correction_) can be calculated by the following formula:

6 $$y_{correction} = \frac{y}{\cos \left( \alpha \right)}.$$

These formulae can be combined into the following formula:

7 $$y_{correction} = \frac{{d_{cal} \pm \left( {\sin \left( \alpha \right) \times i_{cal} } \right)}}{\cos \left( \alpha \right)}.$$

The output of the a.p. radiograph is the distance of the ECM over the hip plane (*d*_*cal*_). By substracting ${y}_{correction}$ from *H*_*ECM*_ (Formula 8) the height of the hip plane above the detector plane can be derived (*H*_*Hip*_).

8 $$H_{Hip} = H_{ECM } - y_{correction}$$

The calibration of any (flat) object or plane of known height above the detector plane can be calculated the intercept theorem in Formula (9):

9 $$Calibation\,Factor = \frac{100 \times S}{{\left( {S - height of object} \right)}}.$$

Therefore, the calibration factor of the hip plane (*F*_*Hip*_) is calculated by:

10 $$F_{Hip} = \frac{100 \times S}{{\left( {S - H_{Hip} } \right)}}.$$

The combined formula is therefore:

11 $$F_{Hip} = \frac{100 \times S}{{S - H_{ECM } - \frac{{d_{cal} \pm \left( {\sin \left( \alpha \right) \times i_{cal} } \right)}}{\cos \left( \alpha \right)} }}.$$

### Algorithm for the correction of patient rotation and lateral offset

In Fig. 9, the algorithm for the correction of rotation and lateral offset and calculation of F_Hip_ is presented.

### Simulation of rotation and lateral offset on F_Hip_

In a standard model, reasonable values were entered based on clinical experience and published literature. Settings were applied specified in Table 3.

**Table 3:** Variables for standard model.

Radiograph	Definition	Variable	Empirical mean value [mm]
lateral x-ray	ECM radius	*Radius*	12.5
	Long half-axis ECM diameter	*b*	35
	Distance Image-center to ECM center	*a*	200
	Horizontal distance right hip center to left hip center	*c*	Simulated
	Distance Horizontal hip center to ECM center	*d*	110
a.-p. x-ray	ECM radius	*Radius*	12.5
	Direct distance Image-center to ECM center	*h*	10
	Horizontal distance Image-center to ECM center (i.e. lateral offset)	*i*	Predifined
	Long half-axis ECM diameter	*k*	35
	Distance right hip center to left hip center	*m*	150

The simulation was informed with rotation in 1° increments from 0° to 30°. Scenarios with lateral offset of 0, 20 and 60 mm were created. *S* is set at 1150 mm. The impact of rotation and lateral offset on *F*_*Hip*_ as difference from the non-rotated value was calculated as absolute deviation and relative deviation.

### Ethical approval

No ethics committee approval was required for this type of study. No clinical data was included. No research was performed on humans or animals.

## Results

### Simulation of patient rotation effects

The predefined settings resulted in a calibration factor of *F*_*Hip*_ = 123.45. Results for rotation and lateral offset dependent simulation of *F*_*Hip*_ with otherwise unchanged variables are presented in Table 4.

**Table 4:** Results of simulation.

	Absolute error			Relative error		
Rotation (°)	i = 0	i = 20	i = 60	i = 0	i = 20	i = 60
0	0.000	0.000	0.000	0.0%	0.0%	0.0%
1	0.002	0.048	0.141	0.0%	0.0%	0.1%
2	0.009	0.101	0.286	0.0%	0.1%	0.2%
3	0.020	0.159	0.435	0.0%	0.1%	0.4%
4	0.036	0.221	0.589	0.0%	0.2%	0.5%
5	0.056	0.287	0.747	0.0%	0.2%	0.6%
6	0.080	0.358	0.909	0.1%	0.3%	0.7%
7	0.109	0.433	1.076	0.1%	0.4%	0.9%
8	0.143	0.514	1.248	0.1%	0.4%	1.0%
9	0.181	0.599	1.424	0.1%	0.5%	1.2%
10	0.224	0.688	1.606	0.2%	0.6%	1.3%
11	0.272	0.783	1.792	0.2%	0.6%	1.5%
12	0.325	0.883	1.983	0.3%	0.7%	1.6%
13	0.382	0.987	2.180	0.3%	0.8%	1.8%
14	0.445	1.097	2.382	0.4%	0.9%	1.9%
15	0.512	1.212	2.589	0.4%	1.0%	2.1%
16	0.585	1.333	2.802	0.5%	1.1%	2.3%
17	0.662	1.459	3.021	0.5%	1.2%	2.4%
18	0.746	1.591	3.246	0.6%	1.3%	2.6%
19	0.834	1.728	3.477	0.7%	1.4%	2.8%
20	0.928	1.871	3.714	0.8%	1.5%	3.0%
21	1.028	2.021	3.958	0.8%	1.6%	3.2%
22	1.134	2.177	4.209	0.9%	1.8%	3.4%
23	1.246	2.339	4.466	1.0%	1.9%	3.6%
24	1.364	2.508	4.731	1.1%	2.0%	3.8%
25	1.489	2.683	5.003	1.2%	2.2%	4.1%
26	1.620	2.866	5.283	1.3%	2.3%	4.3%
27	1.758	3.056	5.571	1.4%	2.5%	4.5%
28	1.903	3.254	5.867	1.5%	2.6%	4.8%
29	2.055	3.459	6.172	1.7%	2.8%	5.0%
30	2.215	3.673	6.486	1.8%	3.0%	5.3%

Error is defined as difference of calculated calibration factor and true calculation factor. *i* is the lateral offset of the marker (i.e. displacement of the marker) in mm. Rotation in degree is the simulated patient rotation in lateral radiographs.

The clinically relevant absolute error of 1 was reached at 21° rotation without lateral offset, 13° with 20 mm lateral offset and 7° with 60 mm offset. The absolute error with possible impact on implant choice (i.e. 1.5%) was reached at 26°, 18° and 10° in each offset setting. The absolute errors reached 2.2, 3.6 and 6.4 at 30° rotation for each offset setting, respectively.

**Figure 10:**
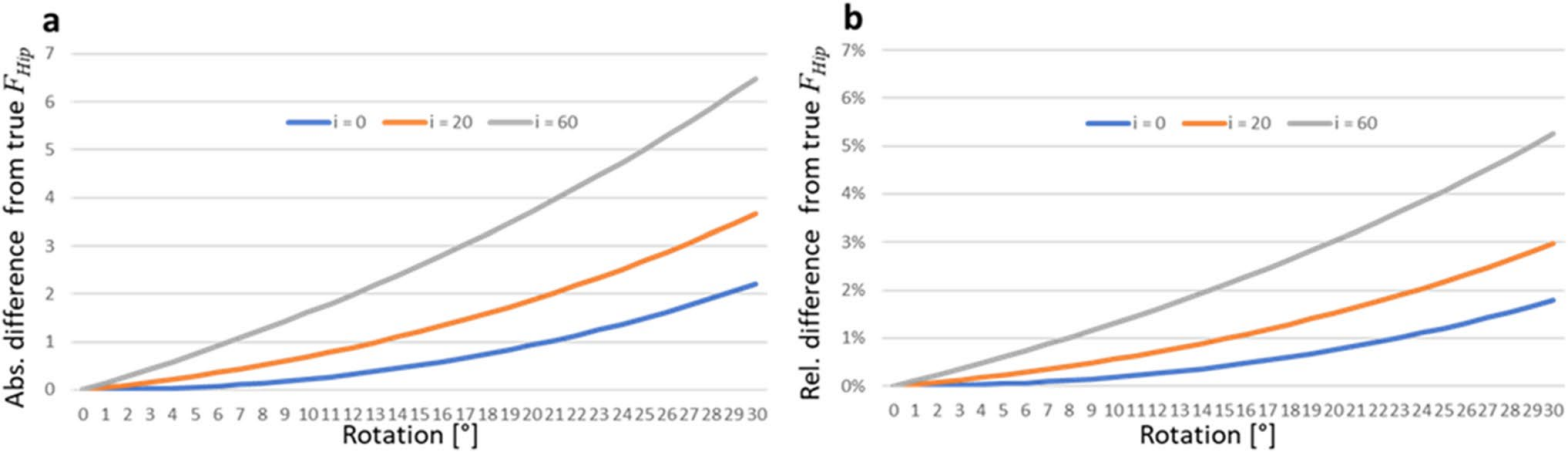
The three lines demonstrate three settings of lateral offset (*i*) in mm. (**a**) absolute error of *F*_*Hip*_ resulting from rotation. (**b**) relative error in percent of *F*_*Hip*_ resulting from rotation.

The errors are shown in Fig. 10.

## Discussion

Quality and risk management require pre-operative templating for THR. Adequate image calibration of radiographs is an essential prerequisite for templating^8,9^. Current methods have been shown to be unreliable and lack sufficient precision. The novel bi-planar calibration method presented here may be the first non-tomography-based calibration method to allow for precise and patient-individual calibration of radiographs.

The standard for digital templating in hip surgery is the use of antero-posterior radiographs of the pelvis and additional planes including axial images of the proximal femur and often lateral imaging for spino-pelvic parameters^17^. Therefore, the novel method does not require additional radiographs or additional equipment. Standard marker balls and fixation devices may be used.

The error of calibration is defined by the difference from the reference calibration factor of the hip plane. It can be tested in radiographs with implants of known size in the region of interest (e.g., THR implants) as reference. Following a recent publication on effects of calibration errors on implant selection, an error equal to or below 1.5% is negligible^16^. Above 1.5%, a deviation of about one implant size per 3–4% calibration error can be assumed^16^. Therefore, the aim of any calibration method should be a maximum error of 1.5%.

Although current literature reports an error of ± 1 implant size or error of 3% (± 1.5) to be acceptable, a more stringent threshold of 1.5% error aiming for zero component size deviation may be a predicate for future improvements of templating^18^. In particular, integration of templating into a more comprehensive workflow of navigated and robotic-assisted surgery will require a more reliable and more precise templating.

Currently, three methods are considered established in clinical practice. All methods show relevant deviations from the set goal of reliable and valid calibration with an error ≤ 1.5%. The current standard of individual calibration with marker balls has been shown to be unreliable with significant deviations from the reference values. While Franken et al. reported the marker-ball method to achieve a mean error of 2% with a maximum error of 6.5%, these results were not reproducible by other authors^9^. Sinclair found a mean error of 6.8% (max. 26%) and Bayne of 8.9%^5^. Boese and Loweg et al. reported on errors in four studies with mean errors of 5.2%, 5.4%, 10.3% and 12.5%; the maximum errors were 23.3%, 26.5%, 28,6% and 23.3%, respectively^4,6–8,10,14^. There was a consensus in the literature, that marker-ball calibration is unreliable. Additionally, Sinclair, Boese and Loweg et al. found the historical fixed calibration method to be more reliable that the marker ball method.

For the fixed calibration factor method, the same calibration factor is used for all radiographs. This fixed factor can be a predefined, empirical or be based on markers that are always placed in the same place, resulting in a consistent calibration factor. Per definition, these fixed factors have an inherent error by not being individual. The known variance of the hip plane position above the detector is ignored and errors are taken into account^6^. Boese et al. found the absolute mean error of the fixed method to be 2.0% (maximum 12.1%)^14^ and Loweg et al.^4^ 1.6% (maximum 8.2%). Similarly, in a simulation of 398 computed tomography (CT) scans, the error was 2.4% (maximum 6.4%)^10^.

The dual-calibration marker method introduced by King et al. combines empirical CT data with marker-based measurement of the sagittal patient diameter^4,10,11^. More recent advancements have reduced the dual-calibration method to one marker in the same position as in the bi-planar calibration method presented herein^4,10^. While Loweg et al.^4^ were able to show superiority of the method over the marker-ball method as well as the fixed method in the supine position, there were still 45% of cases with an error above 1% (absolute mean error 1.1%, maximum 5.6%). Data for standing radiographs was able to show superiority of the dual-scale method over marker balls but not over the fixed calibration method. The mean error was 2.2% (maximum 9.5%)^14^. Overall, these reports consistently show the lack of a sufficiently precise and reliable method for calibration and a need for better methods.

In regards of bi-planar imaging, the EOS system has recently been proposed for marker-based calibration. While this method is similar to the proposed novel approach, it requires the EOS system and thus adds significant cost and complexity^19^. Still, the proposed method can be combined with the EOS system or similar devices.

The theoretical analysis of patient rotation on the bi-planar method demonstrated a maximum error for *F*_*Hip*_ of 6.5% for a rotation of 30° in lateral radiographs with 60 mm lateral offset. In clinical reality, adequate patient positioning should be possible with a maximum of 10–15° rotation and less. It can be assumed that significantly rotated images above 15° rotation are identified as such by anatomical landmarks. Additionally, the error of rotation affects only one part of the method. The overall impact of this error has been considered in a simulation of a standard model based on empirical data. Here, a rotation of 30° would result in a calibration error of 1.4%. This is within the set benchmark for acceptability stated above. In a realistic setting with rotation of up to 15°, the overall calibration error of 0.36% is negligible. While a more pronounced effect of rotation is possible in non-standard model combination of variables, it may be safe to assume negligible effects in alternative settings.

Following the description of the method, a proof of concept study in a laboratory setting is recommended before a clinical application can be considered. While calibration is a key step and predominant source of error in templating, various factors may add to errors in templating. Besides image quality, anatomical variations and precision of measurements, intra-operative decision-making may require deviations from a template. Reasons may include but are not limited to: surgical technique, tissue management (e.g. reaming), and bone quality.

### Limitations

There are limitations to the novel method. The key limitation of rotational errors due to patient positioning has been analyzed and discussed above. Additionally, use errors may result in misplacement of the marker in relation to the pubic symphysis. However, the identification of the anatomical landmark is part of standard setting of the central beam and should be assumed to be fairly reliable. Deviations from the central beam are corrected by the applied formula and similar to the rotational influence only have negligible effects. Due to the bi-planar depiction, a grossly misplaced marker would be identified as such. Another possible user-error is insufficient calibration of marker-ball distance from central beam in either or both radiographs. Single calibration with the known formula may result in overestimation of the projected distance from the central beam. Therefore, the application repeated corrections are recommended. Three (automated) repetitions render any effect clinically negligible.

## Conclusions

The novel bi-planar calibration method may provide the first reliable and adequate calibration of the patient-individual hip plane for total hip replacement and reconstructive surgery. Patient rotation has been identified as possible key limitation with negligible effects on calibration factors for up to 30° of rotation. Clinical studies are now needed to confirm the reliability and validity of the proposed method.

## Data Availability

Data is stored on file at the University Medical Center Hamburg-Eppendorf. CKB should be contacted if necessary.

## Code availability

Not applicable. The underlying mathematical models have been published and are available in the cited articles.
